# Response shift in patient-reported outcomes: definition, theory, and a revised model

**DOI:** 10.1007/s11136-021-02846-w

**Published:** 2021-04-28

**Authors:** Antoine Vanier, Frans J. Oort, Leah McClimans, Nikki Ow, Bernice G. Gulek, Jan R. Böhnke, Mirjam Sprangers, Véronique Sébille, Nancy Mayo, the Response Shift - in Sync Working Group

**Affiliations:** 1 Inserm - University of Nantes - University of Tours, UMR 1246 Sphere “Methods in Patient-Centered Outcomes and Health Research”, Nantes, France; 2 University Hospital of Tours - Inserm, CIC 1415, Unit of Methodology Biostatistics and Data-Management, Tours, France; 3 University of Amsterdam, Research Institute of Child Development and Education, Amsterdam, The Netherlands; 4 Department of Philosophy, University of South Carolina, Columbia, SC USA; 5 Center for Outcomes Research and Evaluation, McGill University, Montreal, Canada; 6 Harborview Medical Center, University of Washington, Seattle, WA USA; 7 College of Nursing, Washington State University, Spokane, WA USA; 8 School of Health Sciences, University of Dundee, Dundee, UK; 9 Department of Medical Psychology, Location AMC, Research Institute Amsterdam Public Health, Amsterdam University Medical Centers, Amsterdam, The Netherlands; 10 Unit of Methodology in Clinical Research and Biostatistics, University Hospital of Nantes, Nantes, France; 11 Division of Clinical Epidemiology, Department of Medicine, McGill University Health Centre Research Institute, Montreal, Canada; 12 Inserm U1246 Sphere, Institut de Recherche en Santé 2 - Université de Nantes, 22, Boulevard Bénoni-Goullin, 44200 Nantes, France

**Keywords:** Response shift, Patient-reported outcomes, Quality of life, Definition, Theory, Model, Psychometrics

## Abstract

**Purpose:**

The extant response shift definitions and theoretical response shift models, while helpful, also introduce predicaments and theoretical debates continue. To address these predicaments and stimulate empirical research, we propose a more specific formal definition of response shift and a revised theoretical model.

**Methods:**

This work is an international collaborative effort and involved a critical assessment of the literature.

**Results:**

Three main predicaments were identified. First, the formal definitions of response shift need further specification and clarification. Second, previous models were focused on explaining change in the construct intended to be measured rather than explaining the construct at multiple time points and neglected the importance of using at least two time points to investigate response shift. Third, extant models do not explicitly distinguish the measure from the construct. Here we define response shift as an effect occurring whenever observed change (e.g., change in patient-reported outcome measures (PROM) scores) is not fully explained by target change (i.e., change in the construct intended to be measured). The revised model distinguishes the measure (e.g., PROM) from the underlying target construct (e.g., quality of life) at two time points. The major plausible paths are delineated, and the underlying assumptions of this model are explicated.

**Conclusion:**

It is our hope that this refined definition and model are useful in the further development of response shift theory. The model with its explicit list of assumptions and hypothesized relationships lends itself for critical, empirical examination. Future studies are needed to empirically test the assumptions and hypothesized relationships.

**Supplementary Information:**

The online version contains supplementary material available at 10.1007/s11136-021-02846-w.

## Introduction

*Patient Reported Outcomes Measures* (PROMs) of *constructs* such as Quality of Life (QoL) are important patient-centered outcomes that are used to evaluate healthcare interventions [1]. Measurement requires standardization to be valid and reliable for estimating change. *Longitudinal measurement invariance* is considered a required condition for allowing comparisons of PROM *scores* over time [2]. The actual occurrence of this condition in the context of analyzing longitudinal PROM data has been challenged [3] and was illustrated by what were initially called “paradoxical and counter-intuitive findings” [4], such as reports of stable or improving QoL over time by patients with a life-threatening disease [5]. Such findings suggest that the *meaning* of some constructs and items is time dependent and patients understand them differently as they go through new life experiences. This suggestion is especially important when the instruments aim to be patient-centered [6, 7]. Evaluation-based self-reports (i.e. self-reports which involve judgment using idiosyncratic criteria such as items like “How difficult is it to walk up a flight of stairs?”) are particularly prone to this change in meaning over time [7]. This phenomenon is now known as *response shift* [3].

In the last 25 years, a growing body of literature has explored the intricacies of considering response shift in measuring constructs [8]. Various definitions and theories were proposed to integrate response shift in explaining change in self-reports [3, 6, 9, 10]. Multiple methods were proposed to analyze response shift in PROM data [11, 12]. Response shift was evidenced in various conditions [13, 14]. These studies have helped to better understand occasional discrepancies between researchers’ or healthcare professionals’ expected assessments of patients’ health and patients’ self-reported health, by highlighting processes such as *psychological adaptation to illness* or the *appraisal* of PROM items. Thus, these insights have enriched the interpretation of PROM results [8, 15]. Meanwhile, fundamental debates continued, evolving around the definition of response shift [16–29], the act of measuring subjective constructs [30], and the relationships between response shift and related concepts [31–34].

Hence, a critical, comprehensive review and synthesis of the work on response shift was deemed crucial. In 2019, an international, interdisciplinary working group of 26 researchers, consisting of response shift experts, new investigators, and independent external experts was formed to achieve this synthesis [14]. They were divided in four teams [12, 14, 15], with the current team focusing on definition and theory.

The objectives are to: (1) outline extant definitions and theories of response shift and related concepts; (2) identify the predicaments encountered in the response shift definitions and theories; (3) propose a more specific, formal definition of response shift; and (4) illustrate it with a revised model addressing the identified predicaments. We also provide some examples of how specific parts of the proposed model can be tested (eText1), while acknowledging that details about operationalizations of model entities are beyond the scope of this paper.

### Extant definitions, theories of response shift and related concepts (Supplementary eTable 1)

The concept of response shift dates back to research on organizational change, where in 1976, Golembiewski proposed a typology of change that took into account that some intervals of a measurement continuum associated with a constant conceptual domain may be recalibrated (*beta change*) and that some domains may be reconceptualized (*gamma change*) [35].

Independent of this work, in the field of education, the term “response shift” was coined by Howard et al. in 1979 as an explanation for an observed discrepancy between quantitative self-reports (an increase in self-reported dogmatism at the group-level after an intervention designed to reduce dogmatism) and qualitative interviews (endorsing that the intervention was considered beneficial) [36]. Howard et al. hypothesized a change in *internal standards of measurement* of dogmatism in people’s mind explaining this discrepancy. They proposed to extend the pretest–posttest research design with a retrospective self-assessment of the pretest level (called “then-test”) immediately administered after posttest assessment. The posttest minus then-test difference was considered a better method of assessing the intervention induced change as both measurements were presumably taken within the same cognitive framework (that from the posttest perspective). Response shift was then defined as the mean difference between pretest and then-test self-report ratings [9].

Sprangers and Schwartz [3] combined and expanded the two aforementioned definitions and proposed a working definition of response shift as *a change in the meaning of one’s self evaluation of a target construct* as a result of three causes. First, *recalibration,* indicating a change in the respondent’s internal standard of measurement. For example, a person may rate his/her chronic back pain level on a Visual Analogue Scale as 5/10 with 10 being the worst pain imaginable. However, after experiencing an extreme acute pain such as renal colic, providing a new experience of the worst pain imaginable, the patient, may rate his pain level as 3/10 despite the level of pain being the same as before. Second, *reprioritization,* which is a change in the importance of component domains constituting the target construct. To illustrate, after a car crash that resulted in permanent motor deficits, social functioning and good relationships can become more important for one’s quality of life than physical functioning. Third, *reconceptualization*, which pertains to a redefinition of the target construct. For instance, after experiencing depressive disorder, mental health may be understood as including components previously related to physical health such as exhaustion. A theoretical model was proposed where a *catalyst* (a salient health event, e.g., initiation of a medical treatment) may trigger *psychological mechanisms* (e.g. coping, social comparison) to accommodate the health change, which in turn may induce response shift that can affect the self-evaluation of the target construct (e.g. QoL) [3]. The kind of mechanisms an individual would adopt and the magnitude and type of response shift that would result, was made dependent on dispositional characteristics that were termed *antecedents*.

In 2004, Rapkin and Schwartz proposed an updated model focusing on the previously insufficient differentiation of response shift from both mechanisms and outcomes. They contend that any self-report is a function of *appraisal* (i.e. the cognitive processes needed for answering survey questions [37]) [6]. Four main types of appraisal processes were specified. Response shift is defined as changes in appraisal (e.g. a change in *standard of comparison* such as comparing pain from “the worst pain I’ve ever had” to “what my doctor told me to expect”), that can account for unexpected changes in QoL that cannot be explained by “*standard influences*” (such as the impact of the catalyst) [6].

In 2005, Oort adopted a different perspective in an attempt to enhance definitional clarity by proposing a formal definition of response shift [10] as a special case of *violation of the Principle of Conditional Independence* (PCI) [38]. Conditional independence refers to the situation where a PROM provides the same results across different samples or over time, given that there are no differences or changes in the target construct. In 2009, Oort and colleagues used this definition to distinguish between two perspectives on response shift. From a *measurement perspective*, response shift occurs when change in the target construct is not fully reflected by the observed change in the measurement. In the *conceptual perspective*, response shift is viewed as an effect occurring when change in the construct is not only explained by “standard influences” (i.e., acknowledged explanatory variables) but also by other variables such as the impact of psychological mechanisms [10].

These laudable attempts to define response shift did not prevent people from attaching diverse meanings to the term [10, 16]. Table 1 lists a range of frequently employed concepts in the literature that are related to response shift. We defined these concepts and clarified their relationships to response shift. For example, in health psychology, post-traumatic growth can be viewed as a cause of response shift. In the context of measurement theory, concepts for which violations of conditional independence are used to identify systematic differences in indicators across time are clearly related to response shift (e.g., when investigating differential item functioning [38] or non-invariance between measurements at different points in time in a longitudinal study [39]). But those are only approaches to detect phenomena that could be the result of a response shift occurring, not necessarily the response shift itself (see Table 1).

**Table 1:** Concepts that are related to but distinct from response shift itself

Related concepts	Definition	Relationship to response shift
Health psychology		
Adaptation	“Modification to suit different or changing circumstances. In this sense, the term often refers to behavior that enables an individual to adjust to the environment effectively and function optimally in various domains, such as coping with daily stressors.” (https://dictionary.apa.org/ adaptation)	Response shift can be a possible effect of adaptation to changing circumstances
Adaptive preferences	“Adaptive preference formation is the unconscious altering of our preferences in light of the options we have available.” [49]	Response shift can be a possible effect of adaptive preference formation
Maladaptation	“A condition in which biological traits or behavior patterns are detrimental, counterproductive, or otherwise interfere with optimal functioning in various domains, such as successful interaction with the environment and effectual coping with the challenges and stresses of daily life.” (https://dictionary.apa.org/maladaptation)	Response shift is rarely associated with maladaptation. Maladaptation is often considered a condition that prevents response shift to occur. We cannot exclude the possibility that maladaptation may also induce response shifts, however, in the opposite direction of adaptation
Coping	“The use of cognitive and behavioral strategies to manage the demands of a situation when these are appraised as taxing or exceeding one’s resources or to reduce the negative emotions and conflict caused by stress.” (https://dictionary.apa.org/coping). Coping styles are generally considered fixed, coping states are considered to respond to a situation. The accommodative mode of coping, for example, is considered a way of neutralizing a situation to make it appear less negative and more acceptable [50]	Response shift can be a possible effect of coping with a taxing situation or negative emotions
(Cognitive) Homeostasis	“Maintenance of a stable balance, evenness, or symmetry.” (https://dictionary.apa.org/homeostasis)	Response shift can both be a possible cause or effect of maintaining or returning to homeostasis, given that this will likely be an iterative process
Health psychology theories	Many theories in health psychology purport to explain how people adapt, cope, and regain balance after a disruptive event. Examples include, but are not limited to: control theories [51], self-regulation theories [52], set-point theories such as adaptation level theories [53, 54], stress-coping theories [55], uncertainty in illness theories [56], discrepancy theories [57], social comparison theory [58], and meaning-making theories [59]	These theories describe possible mechanisms by which adaptation, coping and regaining balance can take place, and in turn may induce response shift
Transformative learning	“…the process of "perspective transformation" has three dimensions: psychological (changes in understanding of the self), convictional (revision of belief systems), and behavioral (changes in lifestyle). Transformative learning is the expansion of consciousness through the transformation of basic worldview and specific capacities of the self …”. (https://en.wikipedia.org/wiki/Transformative_learning#cite_note-1)	Response shift can be a possible effect of transformative learning
Post-traumatic growth or benefit finding	“Positive psychological change experienced as a result of adversity and other challenges to rise to a higher level of functioning. These circumstances represent significant challenges to the adaptive resources of the individual and pose significant challenges to their way of understanding the world and their place in it. Post-traumatic growth involves "life-changing" psychological shifts in thinking and relating to the world, that contribute to a personal process of change, that is deeply meaningful.” (https://en.wikipedia.org/wiki/Posttraumatic_growth)	Response shift can be a possible effect of post-traumatic growth or benefit finding
Post-traumatic depreciation	“The opposite of growth; it is a reduced or impaired sense of psychological adjustment, cognitive development, and emotional awareness” [60, 61]	Response shift is rarely associated with post-traumatic depreciation. Depreciation is often considered a condition that prevents response shift to occur. We cannot exclude the possibility that depreciation may also induce response shifts, however, in the opposite direction of growth
Appraisal	“The cognitive evaluation of the nature and significance of a phenomenon or event.” (https://dictionary.apa.org/appraisal). In the context of Quality of Life (QoL) and response shift, Rapkin & Schwartz [6, 25] narrowed this evaluation down to any response to a QoL item that can be understood as a function of an appraisal process (see supplementary eTable 1)	According to Rapkin & Schwartz [6, 25], changes in appraisal that can explain unexpected changes in QoL, after taking into account standard influences, are response shift. Others suggested that changes in appraisal are causes of response shift rather than response shift itself [27, 29]. In the current paper we also consider changes in appraisal as a possible cause how response shift can occur, i.e., how observed change cannot be fully explained by target change
Recalibration	“In measurement technology … calibration is the comparison of measurement values delivered by a device under test with those of a calibration standard of known accuracy.” (https://en.wikipedia.org/wiki/Calibration). With self-reports, there is no external standard of known accuracy and the respondent is his/her own standard [62]. Recalibration refers to a change in the respondent’s internal standard of measurement [3], which can cause a different interpretation of the response scales over time (e.g., the extremes may become more or less extreme, intervals may change)	According to Sprangers & Schwartz [3], recalibration is one of the three types of response shift. According to Mayo [27] and in the current paper, recalibration is one of the possible causes of how response shift can occur
Reprioritization	“Change in the respondent’s values, i.e., the importance of component domains constituting the target construct.” [3]	According to Sprangers & Schwartz [3], reprioritization is one of the three types of response shift. According to Mayo [27] and in the current paper, reprioritization is one of the possible causes of how response shift can occur
Reconceptualization	“Redefinition of the target construct.” [3]	According to Sprangers & Schwartz [3], reconceptualization is one of the three types of response shift. According to Mayo [27] and in the current paper, reconceptualization is one of the possible causes of how response shift can occur
Implicit theory of change	According to this theory in recalling past states, a two-step process takes place: (1) The current state of attribute or belief is assessed; (2) A theory of stability or change is invoked. From the combination of these two steps the earlier state of attribute or belief is inferred. This theory suggests that recollection of past states would be biased if a person's state has changed but they expect no change to have occurred, or vice versa [63]. For example, if a change in skill is expected, but there is no actual improvement, people will believe that their past skill state was worse than it was [64]	Implicit theory of change is an alternative explanation of response shift [30] that need to be ruled out or controlled for, particularly for those response shift methods that require recall of past events, including the then-test, individualized methods, e.g., the SEIQoL, and qualitative methods (see also recall bias)
Measurement theory and psychometrics		
Measurement bias	Any differential or systematic difference between scores of different groups on a test. “Generally speaking, measurement bias can be said to occur if the test differentially denotes the target construct across different groups or if the nature of the construct assessed by the test differs across the groups” [65]. Measurement bias is not limited to simultaneous testing but can also occur in testing over time [65]	Response shift can be considered a special case of measurement bias when target change is not fully explained by observed change
Differential item functioning (DIF)	“A statistical characteristic of an item that shows the extent to which the item might be measuring different abilities for members of separate subgroups. […] An item … displays DIF if and only if people from different groups with the same underlying true ability have a different probability of giving a certain response.” (https://dictionary.apa.org/measurement-invariance). This characteristic is also referred to as item bias [38]. Conceptually, DIF can be considered a special case of measurement bias (see above); and from a measurement perspective it is a special case of violation of the Principle of Conditional Independence	Response shift can be considered a special case of longitudinal DIF [39], when a discrepancy between observed change and target change occurs
Measurement invariance	“The situation in which a scale or construct provides the same results across several different samples or populations.” (https://dictionary.apa.org/measurement-invariance). For example, a generic QoL questionnaire could be said to have measurement invariance if it yields similar results for individuals of varying gender, age, or disease. Invariance does not have to be limited to invariance across groups, but also over time. Other factors can also be ‘violators’ of measurement invariance [66]. Conceptually, violations of measurement invariance can be considered as a special case of measurement bias (see above); and from a measurement perspective is a special case of violation of the Principle of Conditional Independence	Response shift can be considered a special case of violation of longitudinal measurement of invariance, when a discrepancy between observed change and target change occurs
Measurement error	Any non-systematic difference between a test score and the true score or latent variable. Measurement error may arise from flaws in the questionnaires, mistakes in the administration of the questionnaire, or chance factors. For example, an investigator may obtain biased results from a survey because of problems with wording of response options or variability in administration (https://dictionary.apa.org/measurement-error)	Response shift is not measurement error as it is a systematic and not a random phenomenon
Response bias	The “systematic tendency to respond to a range of questionnaire items on some basis other than the specific item content” [67]. Among the more common types of response tendencies are acquiescence bias (agreement or acceptance, typically without protest or argument), demand characteristics (contextual cues that may influence or bias participants’ responses; https://dictionary.apa.org/demand-characteristics), midpoint or extreme responding, and social desirability bias [68]	As response bias is specifically defined as independent of the item content [68], it is distinct from response shift, which necessitates different interpretations of the meaning of item content. It may therefore be an alternative explanation for response shift effects that need to be ruled out or controlled for
Framing effect	Different ways of presenting the same information lead to different responses, emotions, decisions, or behavior. To exemplify, a medical treatment will be accepted more readily if it is presented positively as the chance of survival (e.g., 90%) than when it is presented negatively as the chance of death (e.g., 10%) [69]. (https://en.wikipedia.org/wiki/Framing_effect_(psychology)	Framing effect can cause a violation of measurement invariance if the presentation is not held constant over time. Framing effects may then be considered an alternative explanation of response shift that needs to be ruled out
Order effect	Different order of presenting the same information lead to different responses, emotions, decisions, or behavior. To exemplify, items A and B will be differently perceived and responded to depending on their order of presentation (A-B or B-A) in a questionnaire. The order effect might not only affect the answer to A but also the association between A and B [70]	Order effect can cause a violation of measurement invariance if the order of presentation is not held constant over time. Order effects may then be considered an alternative explanation of response shift that needs to be ruled out
Practice effect	Any change or improvement that results from practice or repetition of task items or activities. The practice effect is of particular concern in experimentation involving within-subjects designs, as participants’ performance on the variable of interest may improve simply from repeating the activity rather than from any study manipulation imposed by the researcher (https://dictionary.apa.org/practice-effect)	Practice effect can lead to an overestimation of within-subjects change of a target construct. It can also cause a violation of measurement invariance and then be considered an alternative explanation of response shift that needs to be ruled out
Anchoring effects	Presentation of different initial pieces of information (the ‘anchor’) while maintaining the same content lead to different responses, emotions, decisions, or behavior. To exemplify, responses to items A and B might be different when they follow either item X (A, B) or item Y (A, B). (https://en.wikipedia.org/wiki/Anchoring_(cognitive_bias))	Anchoring effects can cause a violation of measurement invariance if the anchors are not held constant over time. Anchoring effects may then be considered an alternative explanation of response shift that needs to be ruled out
Recall bias	A systematic error that often occurs when an individual reports a past behavior or event. Such retrospective reporting may tend to include inaccurate aspects, such as a systematic underestimation or overestimation of the frequency with which a certain behavior occurred (https://dictionary.apa.org/recall-bias).	Recall bias is relevant to response shift methods that require retrospection (i.e., the then-test, individualized measures as the SEIQoL, and qualitative interviews [12]. Howard et al. [36] consider recall bias as an alternative explanation of response shift that needs to be ruled out. Conversely, Collins et al. [71] consider recall bias an indication of response shift: If response shift has occurred, respondents use the response scale differently and are therefore not able to recall their previous ratings

### Predicaments encountered in previous definitions and theories of response shift

Several predicaments were encountered during the review of the definitions and theories of response shift. First, in an attempt to reconcile different perspectives on response shift, Oort et al. proposed two definitions of response shift, from the measurement and the conceptual perspective [10]. Each definition was formulated using the same (statistical) terminology, i.e., as a violation of conditional independence. However, this distinction has not been widely adopted, possibly on account of a too general conceptualization, encompassing other instances of measurement bias and its statistical foundation may have been too complex. We therefore propose further specification and clarification of their response shift definition.

Second, as response shift is a time-dependent phenomenon related to change, the models of Sprangers and Schwartz [3] and Rapkin and Schwartz [6] are indeed focused on explaining change in the target construct. For example, in the Rapkin and Schwartz model, the processes are shown to drive “Change in Quality of Life” [6]. By focusing on explaining change in the target construct rather than explaining the construct at each measurement occasion (with at least two time points as the simplest model), those models neglected the importance of using multiple time points to investigate response shift. Incorporating at least two time points in a theoretical model would enable a clearer explication of the chain of causality among the constituting components over time [3, 10].

Third, extant models do not explicitly discriminate the target construct (e.g., QoL) from its measure (e.g., PROM). Whereas the construct and its measure are closely related, by definition, response shift is a phenomenon addressing changes in their relationship. Explicitly distinguishing the construct and its measure enables better characterization of how response shift can occur.

### A more specific formal definition of response shift

Usually, a PROM is designed to measure a construct defined with an a priori conceptual model of its component domain(s) and is used after it has been shown to yield *sufficient psychometric quality* [1, 40]. The interpretability and validity of a PROM lies, in part, in ensuring that patients understand the items in the same way the designers intended. However, as answering a PROM inherently involves a subjective process of appraisal [6, 37], a *discrepancy* can occur between the meaning inferred from this process and the meaning the designer wanted to convey. If respondents understood the items in the same way over time (intra-individual invariance of meaning over time), there would be no response shift [34]. But circumstances may change, and that change may impact patients’ interpretations of the item(s). When that happens, it seems reasonable to assume the a priori relationship between the target construct and its measure also has changed over time. Thus, a formal definition of response shift should encompass the occurrence of this discrepancy between measurement occasions.

To address the first predicament, we consider the *measurement perspective* to response shift only. Response shift is then the effect that occurs when circumstances cause people to change their interpretation of the measurement of the underlying target construct, e.g., as the result of accommodating a health change. Consequently, there is a discrepancy between the observed change (e.g., change in PROM scores) and the target change (i.e., change in the target construct). Response shift therefore can be more narrowly defined as a special case of violation of the PCI *when observed change is not fully explained by target change*. This definition can lead to the operationalization of response shift at group level as well as individual level. Moreover, we assume this phenomenon to be the consequence of “*a change in the meaning of one’s self evaluation of a target construct,*” which phrase was used in the working definition of response shift [3].

A possible translation into mathematical terms of the definition (i.e. formal definition) at a statistical level is given by: *there is response shift if ψ*_*1*_*(Observed Change|Target Change) ≠ ψ*_*2*_*(Observed Change|Target Change, Other Variables)*, where ψ_1_ signifies the distribution of observed changes (Observed Change; e.g., change in PROM scores) conditional on the change in the construct intended to be measured (Target Change; e.g., change in QoL), is unequal to ψ_2_, the distribution of Observed Change given change in the target construct and any Other Variables (e.g., adaptation to or coping with a new health state).

This more specific definition considers response shift as an effect but does not explain how this effect occurs. In the context of health care, we need a theoretical model depicting the components that can be understood as “Observed Change”, “Target Change” and especially “Other Variables” (e.g., catalyst, mechanisms, antecedents). Moreover, the model needs to illustrate the relationships between these components over time to unravel the potential pathways leading to response shift. Thus, the next step is to propose a model depicting these components and their relationships at two time points (addressing the second predicament), distinguishing both the target construct and its measure (addressing the third predicament).

### A revised response shift model (Fig. 1, Tables 2 and 3)

**Fig. 1:**
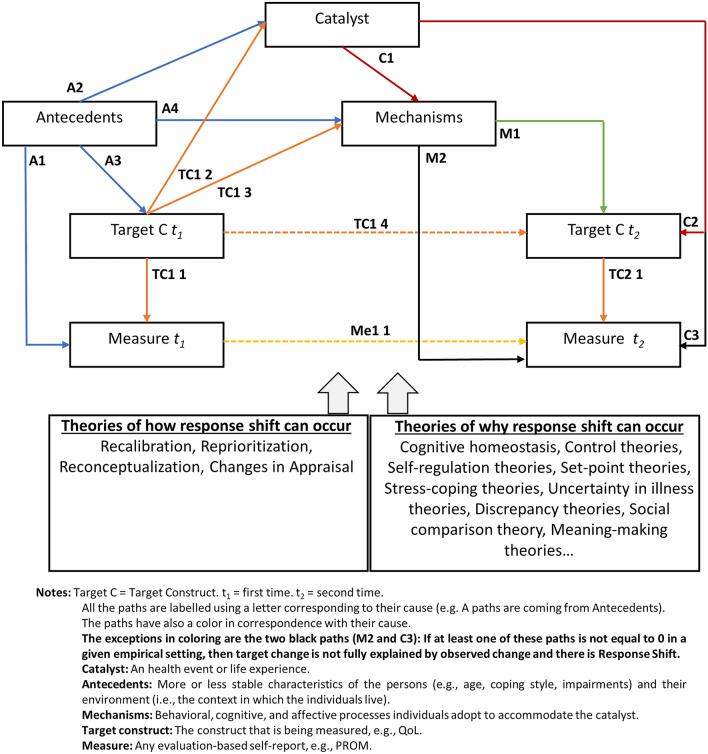
Revised response shift model for evaluation-based self-report data at two time points. Response shift is an effect that occurs through pathways M2 and C3

**Table 2:** Assumptions Underlying the Revised Response Shift Model

A. Epistemic assumptions
A1. The target construct and “other variables” (catalyst, antecedents, mechanisms, measure) are conceptually distinct
B. Methodological assumptions
B1. The measure pertains to an evaluation-based self-report B2. The measure results from the responses to the items as well as from the algebraic transformation applied to derive an estimate of it. As this algebraic transformation is the same at each time, it cannot cause different results over time
B3. The items of the measure are free from poor or ambiguous wording
B4. The measure is reliable and valid
C. Practical assumptions
C1. The catalyst is sufficiently impactful to influence the respondents’ perspective on the measure, either directly or via its influence on mechanisms
C2. Some antecedents may influence the respondents’ perspective on the measure at each time point C3. Some mechanisms may influence the respondents’ perspective on the measure
C4. Response shift occurs in change processes and requires therefore at least two time points
C5. The target construct can be anything that can be measured with evaluation-based self-report

**Table 3:** Outline of the indicated paths in the model (see Fig. 1)

Paths coming from the Antecedents
A1: Antecedents at time 1 may affect the response to the measure at each time point (note: only the line to the measure at time 1 is depicted as the effect at time 2 is carried through Me1 1)
A2: Antecedents may influence the occurrence of a catalyst. For example, a genetic predisposition and smoking history (antecedents) may cause lung cancer (the catalyst)
A3: Antecedents may influence the level of the construct at each time point (note: only the line to the construct at time 1 is depicted as the effect at time 2 is carried through TC1 4)
A4: Antecedents may influence the mechanisms after the catalyst has triggered them (C1). For example, because of personality traits, someone will tend to adapt in a certain way
Paths coming from the target construct
TC1 1 and TC2 1: The target construct explains (in part) the value of the measure
TC1 2: The target construct at time 1 may influence the occurrence of the catalyst. For example, a high level of fatigue (the target construct) may cause a car crash (the catalyst)
TC1 3: The target construct at time 1 may induce mechanisms. For example, a high level of fatigue (the target construct) may induce seeking support (mechanisms)
TC1 4: The target construct at time 1 influences, in part, the target construct at time 2
Path coming from the measure
Me1 1: The measure at time 1 may influence the measure at time 2. This path would correspond to the correlation between residual factors (i.e., all that is specific to the measures plus random error variation)
Path coming from the catalyst
C1: The catalyst triggers mechanisms to adapt to the change in health state
C2: The catalyst may influence the level of the construct at time 2. This is usually the main effect of interest of many studies (e.g. how a certain diagnosis affects QoL)
C3: The catalyst may directly influence the measure’s results at time 2. If this path is not equal to zero, then observed change cannot be fully explained by target change and there will be response shift
Paths coming from the mechanisms
M1: The mechanisms may influence the level of the target construct at time 2. For example, as a result of seeking support, an individual may experience less fatigue at time 2
M2: The mechanisms may influence the time 2 measure’s results. If the C1 (effect of catalyst on mechanisms) path is not equal to zero, the catalyst impacts the measure at time 2, mediated by the mechanisms (C1 then M2 paths) and observed change will not be fully explained by target change, and there will be response shift

The proposed model is a modified version of previous ones [3, 6]. This model makes an explicit distinction between the target construct (e.g., QoL) and its measure (e.g., PROM scores) and shows the conceptual components and their interrelationships at two points in time. It depicts the simplest longitudinal design but can be extended to more time points. It is a *Structural Equation Metamodel* [41], which means it depicts relationships between conceptual entities without any assumptions about the operationalization of such entities as variables or the mathematical form of the relationships among the entities. As the passage of time drives the relationships between entities, cause-effect relationships are proposed. The most plausible paths are depicted and explicitly labeled. Table 2 lists the underlying assumptions of the proposed model.

In addition to the target construct and its measure at each time point, three main interrelated components that featured in the previous models are also retained. First, the model is centered on a *catalyst:* a health event or life experience that can have an impact on the target construct (*C*_2_ path) at time 2. It can differ from person to person, it can be a distinctive event (e.g. a car accident), multiple events happening in a short period of time (e.g. diagnosis of cancer) or experience accumulated with passage of time. The catalyst represents the *necessary* condition leading to change.

Second, *antecedents* are more or less stable characteristics related to personal (e.g., personality, comorbid conditions) or environmental factors (e.g., access to health care) that determine the context in which individuals live (see Fig. 1). Hence, the term is used more broadly, also encompassing environmental factors, than Sprangers and Schwartz did [3]. Several models have been proposed to classify these factors including the International Classification of Functioning, Disability and Health [42] and the Wilson-Cleary Model [43]. In a given empirical situation, these antecedents need to be known because they influence the baseline condition, including the possible occurrence of a catalyst (*A*_2_) and the way someone will react to the catalyst (*A*_4_). Moreover, they may influence the target construct (*A*_3_) and the responses to PROM items (*A*_1_) at time 1. These influences can be carried to time 2 through the *TC1*_4_ path (target construct at time 2) and the *Me1*_1_ path (responses to PROM items at time 2).

Third, *mechanisms* are *psychological processes* triggered by the catalyst to accommodate the threat to one’s homeostasis. These processes may be adaptive or maladaptive and people can adopt more than one mechanism simultaneously to restore the balance (see Fig. 1, and for examples of psychological processes Table 1).

When all the pathways coming from the catalyst, either directly (*C*_2_ and *C*_3_) or mediated by the mechanisms (*C*_1_ then *M*_1_ and *M*_2_ paths), are equal to zero, the variability of the target construct and its measure are carried from time 1 to time 2 (*TC1*_4_, and *Me1*_1_). In that case there is no change. Otherwise, there is change in the construct and/or its measure.

According to this model, response shift occurs when the target construct cannot fully explain the variability of the PROM results at time 2 (another path than the *TC2*_1_ and *Me1*_1_ explain the measure at time 2). Two main pathways indicate the possible occurrence of response shift. First, a direct effect of a catalyst on the PROM at time 2 (e.g. an acute shock due to a near escape from a car accident, influencing the interpretation of a PROM immediately administered afterwards, where the limited passage of time makes the influence of mechanisms less likely). This effect will explain part of the variability in the PROM at time 2 (*C*_3_) and as it is not explained by the variability of the construct (*TC2*_1_), there will be response shift. Second, a more convincing response shift effect occurs when the catalyst impacts the PROM at time 2, mediated by the mechanisms (*C*_1_ then *M*_2_ paths). These paths depict the possibility that *psychological adaptation* to a situation can impact the way someone answers PROM items at time 2. Again, if this influence directly explains, in part, the variability of the PROM at time 2 (*M*_2_), then response shift has taken place.

Finally, apart from its baseline value (*TC1*_1_) and the impact of the catalyst (*C*_2_), the target construct at time 2 can be explained by another pathway: the direct influence of mechanisms (*M*_1_). Nonetheless, we do not consider this as response shift because it impacts the target construct but not the PROM, so it will not lead to a discrepancy between observed and target change. A description and illustration of the individual paths in the model is presented in Table 3.

Response shift and the operational model are not just “armchair” phenomena and processes but refer to real life experiences of people as they go through and try to make sense of health changes. Each of the components of the model have been experienced by people in their everyday lives. Supplementary eTable 2 presents how people have described these experiences in their own words [44].

### Implications of the formal definition and its application to PROMs at two time points

The more specific formal definition and the revised model clarify that response shift is an effect. The revised model specifically explicates the chain of causality among the constituting components over time and the multiple pathways leading to both direct (i.e., impact of the catalyst) and mediated effects (i.e., by mechanisms) on the PROM indicating response shift. Several implications and assumptions warrant attention.

First, a major implication is that recalibration, reprioritization, and reconceptualization (3 Rs) have been removed from the definition of response shift. These concepts are not necessarily response shift in itself. Rather, they explain *how* response shift can occur, i.e. they add further explanation to the processes depicted by the model. The interaction between a catalyst, antecedents, and mechanisms may cause people to recalibrate the measurement scale they need to complete, reprioritize domains they value, and/or reconceptualize the underlying construct they need to rate, such that it will lead to a discrepancy between target change and observed change, hence a response shift.

Similarly, we also consider change in appraisal as an explanation of *how* response shift occurs [27] rather than response shift itself [25]. At each measurement occasion, appraisal is needed to arrive at a response to PROM item(s) [6, 37]. Appraisals are cognitive processes that come into play when a respondent evaluates him/herself with respect to a target construct and chooses a response option. When there is a change in appraisal then the meaning of the observed response changes. Rapkin and Schwartz showed how each of the four appraisal processes they adopted correspond with the 3 Rs [25]. The 3 Rs can thus be viewed as examples of changes in appraisal. It should be noted that changes in appraisal may not be limited to the 3 Rs as more cognitive processes have been identified [37].

Third, in the model we depicted an extra box referring to theories that may explain *why* response shift could occur. These theories purport to explain why people adapt, cope, and try to regain balance after a disruptive event (see Table 1). These theories describe possible mechanisms that may induce response shift and can be considered the underlying theories explaining the main principles behind the model.

The proposed model delineates the plausible paths explaining both changes in the target construct between two times of measurement and offers numerous opportunities for strong predictions and empirical tests. We have adopted an agnostic approach, i.e., we have not specified how the depicted entities are operationalized nor how these are mathematically linked. At the stage of analyzing data, careful attention is needed for appropriate testing of response shift. For example, the target construct can be operationalized as a latent variable inferred from directly measured variables (e.g. scores) using Structural Equation Modeling, Item Response Theory or Rasch Measurement Theory. As these latent variable models allow to formally specify and estimate the measurement model between the target construct (as latent variable(s)) and the measure (e.g. the items) using a set of equations, a test verifying whether this set of equations can be assumed equivalent at each time of measurement can be seen as a formal test of the violation of the PCI [45–47]. Sébille et al.’s critical review of the literature also demonstrated that there are other response shift methods that also examine discrepancies between target change and observed change [12]. To provide a starting point, a selection of approaches to test specific parts of the proposed model are presented in supplementary eText 1. It should be noted that these are mere examples, without intending to narrow the presented model nor the range of potential statistical or psychometric methods. We anticipate that findings which will either support or refute this revised model will require multiple studies, employing a variety of methods.

As mentioned before, we assume that response shift as defined as a special case of violation of the PCI is caused by “a change in the meaning of one’s self evaluation of a target construct”. Our formal definition has the advantage that it separates response shift from its possible causes. It also separates response shift from its methods of detection. Indeed, any method that could detect violations of the PCI in longitudinal data is able to detect response shift. However, as discussed by Sébille et al. [12], violation of the PCI may be considered a necessary but not a sufficient condition for the occurrence of response shift. That is, violation of the PCI may not always imply change in the meaning of one’s self-evaluation. Hence, if we further restrict the definition of response shift by requiring that it must be caused by a change in the meaning of one’s self evaluation, then alternative explanations need to be ruled out before the conclusion that response shift has occurred is warranted (Table 1).

Lastly, our definition and model rely on multiple epistemic, methodological and practical assumptions (Table 2). In our definition and model, response shift is understood to be an effect that occurs when the construct is not similarly measured over time. Thus, the model treats response shift as a discrepancy between a theoretical model where observed change is fully explained by target change at each time of measurement and what happens in reality. Our definition and model seem to conflict with some of the disability literature. Disability-positive testimonies and the disability pride movement advocate that QoL and functioning with disability can be good. These testimonies make a particular point of emphasizing that mechanisms such as coping transform constructs such as QoL and functioning [48]. Put differently, disability-positive testimonies argue that these constructs are heavily idiosyncratic constructs. This alternative conception can help to recognize our definition and model are deeply connected with the idea of measuring a construct in a quantitative manner and are therefore possibly a better fit for a nomothetic approach of constructs using statistical modeling on empirical quantitative data.

## Conclusion

The main purpose of this effort is bringing clarity and specification to the response shift concept, by proposing a formal definition and applying it to a PROM, before and after the occurrence of a hypothesized catalyst. This yields a model in which response shift effects are distinguished from non-response shift effects. This definition and the model are useful in the further development of response shift theory and in advancing empirical research. The model with its explicit list of assumptions and hypothesized (time order and mediation) relationships lends itself for critical, empirical examination, including refutation [14]. Future studies are warranted to empirically test the assumptions and hypothesized relationships.

## Supplementary Information

Below is the link to the electronic supplementary material.

Supplementary file1 (DOCX 202 kb)

## Data Availability

Not applicable.

## References

[1] de Vet HCW (2011). Measurement in medicine: A practical guide.

[2] Fayers PM, Machin D (2007). Quality of life: the assessment, analysis, and interpretation of patient-reported outcomes.

[3] Sprangers MAG, Schwartz CE (1999). Integrating response shift into health-related quality of life research: A theoretical model. Social Sci Med.

[4] Schwartz CE, Sprangers MAG (1999). Methodological approaches for assessing response shift in longitudinal health-related quality-of-life research. Social Science & Medicine.

[5] Andrykowski M, Brady M, Hunt J (1993). Positive psychosocial adjustment in potential bone narrow transplant recipients: Cancer as a psychosocial transition. Psycho-oncology.

[6] Rapkin BD, Schwartz CE (2004). Toward a theoretical model of quality-of-life appraisal: Implications of findings from studies of response shift. Health and Quality of Life Outcomes.

[7] Schwartz CE, Rapkin BD (2004). Reconsidering the psychometrics of quality of life assessment in light of response shift and appraisal. Health and Quality of Life Outcomes.

[8] Vanier, A., Falissard, B., Sébille, V., & Hardouin, J.-B. (2018). The complexity of interpreting changes observed over time in Health-Related Quality of Life: a short overview of 15 years of research on response shift theory. In *Perceived health and adaptation in chronic disease. Stakes and future challenge* (pp. 202–230). New-York: Routledge

[9] Howard GS, Dailey PR (1979). Response-shift bias: A source of contamination of self-report measures. Journal of Applied Psychology.

[10] Oort FJ, Visser MRM, Sprangers MAG (2009). Formal definitions of measurement bias and explanation bias clarify measurement and conceptual perspectives on response shift. Journal of Clinical Epidemiology.

[11] Sajobi TT, Brahmbatt R, Lix LM, Zumbo BD, Sawatzky R (2018). Scoping review of response shift methods: Current reporting practices and recommendations. Quality of Life Research.

[12] Sébille, V., Lix, L. M., Ayilara, O., Sajobi, T. T., Janssens, C. J. W., Sawatzky, R., and the Response Shift - in Sync Working Group. (2021). *Critical examination of current response shift methods and proposal for advancing new methods*. Accepted (same issue): Quality of Life Research. 10.1007/s11136-020-02755-4 PMC8602164 33595827

[13] Schwartz CE, Bode R, Repucci N, Becker J, Sprangers MAG, Fayers PM (2006). The clinical significance of adaptation to changing health: A meta-analysis of response shift. Quality of Life Research.

[14] Sprangers, M. A. G., Sajobi, T. T., Vanier, A., Mayo, N. E., Sawatzky, R., Lix, L.,and the Response Shift - in Sync Working Group. (2021). Response shift in results of patient-reported outcome measures: A commentary to the Response Shift - in Sync Working Group Initiative. *Quality of Life Research*, Online ahead of print. 10.1007/s11136-020-02747-4 PMC8602228 33481193

[15] Sawatzky, R., Kwon, J.-Y., Barclay, R., Chauhan, C., Franck, L., van den Hout, W., and the Response Shift - in Sync Working Group. (2021). Implications of response shift for micro, meso, and macro healthcare decision making using patient-reported outcomes. *Quality of Life Research*. Accepted (same issue) 10.1007/s11136-021-02766-9 PMC8602130 33651278

[16] Schwartz, C. E., Sprangers, M. A., & Fayers, P. M. (2005). Response shift: you know it’s there, but how do you capture it? Challenges to the next phase of research. In: *Assessing quality of life in clinical trials. 2nd edition*. Oxford: Oxford University Press.

[17] Ubel PA, Peeters Y, Smith D (2010). Abandoning the language of “response shift”: A plea for conceptual clarity in distinguishing scale recalibration from true changes in quality of life. Quality of Life Research.

[18] Sprangers MAG, Schwartz CE (2010). Do not throw out the baby with the bath water: Build on current approaches to realize conceptual clarity. Response to Ubel, Peeters, and Smith. Quality of Life Research.

[19] Reeve BB (2010). An opportunity to refine our understanding of “response shift” and to educate researchers on designing quality research studies: Response to Ubel, Peeters, and Smith. Quality of Life Research.

[20] Boyer L, Baumstarck K, Michel P, Boucekine M, Anota A, Bonnetain F, Auquier P (2014). Statistical challenges of quality of life and cancer: New *****avenues for future research. Expert Review of Pharmacoeconomics & Outcomes Research.

[21] Ubel PA, Smith DM (2010). Why should changing the bathwater have to harm the baby?. Quality of Life Research.

[22] Donaldson GW (2005). Structural equation models for quality of life response shifts: Promises and pitfalls. Quality of Life Research.

[23] Oort FJ (2005). Towards a formal definition of response shift (In Reply to G.W. Donaldson). Quality of Life Research.

[24] Boehnke JR, Skolasky RL, Rutherford C (2019). Introduction to “Advancing quality-of-life research by deepening our understanding of response shift”. Quality of Life Research.

[25] Rapkin BD, Schwartz CE (2019). Advancing quality-of-life research by deepening our understanding of response shift: A unifying theory of appraisal. Quality of Life Research.

[26] Finkelstein JA (2019). Measurement of appraisal is a valuable adjunct to the current spine outcome tools: A clinician’s perspective on the Rapkin and Schwartz commentary. Quality of Life Research.

[27] Mayo NE (2019). Appraisal as a unifying theory of response shift: Continuing the conversation. Quality of Life Research.

[28] Sawatzky R (2019). Relating response shift and cognitive appraisal to measurement validation. Quality of Life Research.

[29] Verdam MGE, Oort FJ (2019). Conceptual and methodological considerations regarding appraisal and response shift. Quality of Life Research.

[30] Norman G (2003). Hi! How are you? Response shift, implicit theories and differing epistemologies. Quality of Life Research.

[31] Stanton AL, Revenson TA, Tennen H (2007). Health psychology: Psychological adjustment to chronic disease. Annual Review of Psychology.

[32] Barclay-Goddard, R., King, J., Dubouloz, C.-J., Schwartz, C. E., & Response Shift Think Tank Working Group. (2012). Building on transformative learning and response shift theory to investigate health-related quality of life changes over time in individuals with chronic health conditions and disability. *Archives of Physical Medicine and Rehabilitation,**93*(2), 214–220. 10.1016/j.apmr.2011.09.010 10.1016/j.apmr.2011.09.010 22289229

[33] McClimans L, Bickenbach J, Westerman M, Carlson L, Wasserman D, Schwartz C (2013). Philosophical perspectives on response shift. Quality of Life Research.

[34] Vanier A, Leplège A, Hardouin J-B, Sébille V, Falissard B (2015). Semantic primes theory may be helpful in designing questionnaires such as to prevent response shift. Journal of Clinical Epidemiology.

[35] Golembiewski RT (1976). Measuring change and persistence in human affairs: Types of change generated by OD designs. The Journal of Applied Behavioral Science.

[36] Howard GS, Ralph KM, Gulanick NA, Maxwell SE, Nance DW, Gerber SK (1979). Internal invalidity in pretest-posttest self-report evaluations and a re-evaluation of retrospective pretests. Applied Psychological Measurement.

[37] Tourangeau R, Rips LJ, Rasinski KA (2000). The psychology of survey response.

[38] Mellenbergh GJ (1989). Item bias and item response theory. International Journal of Educational Research.

[39] Mukherjee S, Gibbons LE, Kristjansson E, Crane PK (2013). Extension of an iterative hybrid ordinal logistic regression/item response theory approach to detect and account for differential item functioning in longitudinal data. Psychological Test and Assessment Modeling.

[40] Reeve BB, Wyrwich KW, Wu AW, Velikova G, Terwee CB, Snyder CF, Butt Z (2013). ISOQOL recommends minimum standards for patient-reported outcome measures used in patient-centered outcomes and comparative effectiveness research. Quality of Life Research.

[41] Grace JB, Schoolmaster DR, Guntenspergen GR, Little AM, Mitchell BR, Miller KM, Schweiger EW (2012). Guidelines for a graph-theoretic implementation of structural equation modeling. Ecosphere.

[42] World Health Organization. (2013). *How to use the ICF. A practical manual for using the International Classification of Functioning, Disability and Health (ICF)*.

[43] Wilson IB, Cleary PD (1995). Linking clinical variables with health-related quality of life. A conceptual model of patient outcomes. JAMA.

[44] Ow, N., Vanier, A., Oort, F. J., McClimans, L., Böhnke, J. R., Gulek, B. G., & Mayo, N. E. (2020). A revised operational model of response shift: Examples from patients’ perspectives. *Quality of Life Research*, *27th International Conference of ISOQOL*, S1–196

[45] Oort FJ (2005). Using structural equation modeling to detect response shifts and true change. Quality of Life Research.

[46] Guilleux A, Blanchin M, Vanier A, Guillemin F, Falissard B, Schwartz CE, Sébille V (2015). RespOnse Shift ALgorithm in Item response theory (ROSALI) for response shift detection with missing data in longitudinal patient-reported outcome studies. Quality of Life Research.

[47] Blanchin M, Guilleux A, Hardouin J-B, Sébille V (2020). Comparison of structural equation modelling, item response theory and Rasch measurement theory-based methods for response shift detection at item level: A simulation study. Statistical Methods in Medical Research.

[48] Barnes E (2016). The minority body: A theory of disability.

[49] Colburn B (2011). Autonomy and adaptive preferences. Utilitas.

[50] Brandtstädter J, Renner G (1990). Tenacious goal pursuit and flexible goal adjustment: Explication and age-related analysis of assimilative and accommodative strategies of coping. Psychology and Aging.

[51] Carver CS, Scheier MF (1982). Control theory: A useful conceptual framework for personality-social, clinical, and health psychology. Psychological Bulletin.

[52] Nerenz R, Leventhal H, Burish TG, Bradley LA (1983). Self-regulation theory in chronic illness. Coping with chronic disease: Research and applications.

[53] Brickman P, Campbell D, Appley MH (1971). Hedonic relativism and planning the good society. Adaptation-level theory.

[54] Diener E (2006). Guidelines for national indicators of subjective well-being and ill-being. Journal of Happiness Studies.

[55] Lazarus R, Folkman S (1984). Stress, apraisal, and coping.

[56] Mishel M (1988). Uncertainty in illness. Journal Nursing Scholarship.

[57] Michalos AC (1985). Multiple discrepancies theory (MDT). Social Indicators Research.

[58] Festinger L (1954). A theory of social comparison processes. Human Relations.

[59] Park CL, Folkman S (1997). Meaning in the context of stress and coping. Review of General Psychology.

[60] Barrington A, Shakespeare-Finch J (2013). Posttraumatic growth and posttraumatic depreciation as predictors of psychological adjustment. Journal of Loss and Trauma.

[61] Baker JM, Kelly C, Calhoun LG, Cann A, Tedeschi RG (2008). An examination of posttraumatic growth and posttraumatic depreciation: Two exploratory studies. Journal of Loss and Trauma.

[62] Vanier A (2016). The concept, measurement, and integration of response shift phenomenon in Patient-Reported Outcomes data analyses.

[63] Ross M (1989). Relation of implicit theories to the construction of personal histories. Psychological Review.

[64] Mayo NE (2017). Dictionary of quality of life and health outcomes measurement.

[65] Lievens F, Reeve CL, Heggestad ED (2007). An examination of psychometric bias due to retesting on cognitive ability tests in selection settings. Journal of Applied Psychology.

[66] Oort FJ (2001). Three-mode models for multivariate longitudinal data. British Journal of Mathematical and Statistical Psychology.

[67] Paulhus, D. L. (1991). Measurement and control of response bias. In *Measures of personality and social psychological attitudes* (pp. 17–59). Elsevier. 10.1016/B978-0-12-590241-0.50006-X

[68] Wetzel E, Böhnke JR, Brown A (2016). Response biases. The ITC international handbook of testing and assessment.

[69] Tversky A, Kahneman D (1981). The framing of decisions and the psychology of choice. Science.

[70] Panter AT, Tanaka JS, Wellens TR, Schwarz N, Sudman S (1992). The psychometrics of order effects. Context effects in social and psychological research.

[71] Collins LM, Graham JW, Hansen WB, Johnson CA (1985). Agreement between retrospective accounts of substance use and earlier reported substance use. Applied Psychological Measurement.

