# Correction to: Response shift in patient-reported outcomes: definition, theory, and a revised model

**DOI:** 10.1007/s11136-021-02890-6

**Published:** 2021-05-27

**Authors:** Antoine Vanier, Frans J. Oort, Leah McClimans, Nikki Ow, Bernice G. Gulek, Jan R. Böhnke, Mirjam Sprangers, Véronique Sébille, Nancy Mayo, the Response Shift - in Sync Working Group

**Affiliations:** 1 Inserm - University of Nantes - University of Tours, UMR 1246 Sphere “Methods in Patient-Centered Outcomes and Health Research”, Nantes, France; 2 University Hospital of Tours - Inserm, CIC 1415, Unit of Methodology Biostatistics and Data-Management, Tours, France; 3 University of Amsterdam, Research Institute of Child Development and Education, Amsterdam, The Netherlands; 4 Department of Philosophy, University of South Carolina, Columbia, SC USA; 5 Center for Outcomes Research and Evaluation, McGill University, Montreal, Canada; 6 Harborview Medical Center, University of Washington, Seattle, WA USA; 7 College of Nursing, Washington State University, Spokane, WA USA; 8 School of Health Sciences, University of Dundee, Dundee, UK; 9 Department of Medical Psychology, Location AMC, Research Institute Amsterdam Public Health, Amsterdam University Medical Centers, Amsterdam, The Netherlands; 10 Unit of Methodology in Clinical Research and Biostatistics, University Hospital of Nantes, Nantes, France; 11 Division of Clinical Epidemiology, Department of Medicine, McGill University Health Centre Research Institute, Montreal, Canada; 12 Inserm U1246 Sphere, Institut de Recherche en Santé 2 - Université de Nantes, 22, Boulevard Bénoni-Goullin, 44200 Nantes, France

## Correction to: Quality of Life Research 10.1007/s11136-021-02846-w

The article “Response shift in patient‑reported outcomes: definition, theory, and a revised model”, written by Antoine Vanier, Frans J. Oort, Leah McClimans, Nikki Ow, Bernice G. Gulek, Jan R. Böhnke, Mirjam Sprangers, Véronique Sébille, Nancy Mayo and the Response Shift - in Sync Working Group, was originally published electronically on the publisher’s internet portal on 28 April 2021 without open access. With the author(s)’ decision to opt for Open Choice the copyright of the article changed on 17 May 2021 to © The Author(s) 2021 and the article is forthwith distributed under a Creative Commons Attribution 4.0 International License, which permits use, sharing, adaptation, distribution and reproduction in any medium or format, as long as you give appropriate credit to the original author(s) and the source, provide a link to the Creative Commons licence, and indicate if changes were made. The images or other third party material in this article are included in the article’s Creative Commons licence, unless indicated otherwise in a credit line to the material. If material is not included in the article’s Creative Commons licence and your intended use is not permitted by statutory regulation or exceeds the permitted use, you will need to obtain permission directly from the copyright holder. To view a copy of this licence, visit http://creativecommons.org/licenses/by/4.0.

The original article has been corrected.

