# Supplementary material for: Response shift in patient-reported outcomes: definition, theory, and a revised model
Source: Qual Life Res. 2021 Apr 28;30(12):3309–22. doi: 10.1007/s11136-021-02846-w (PMC8602159; doi:10.1007/s11136-021-02846-w)
Supplement: Supplementary file 1 — Supplementary file1 (DOCX 202 kb) [file 11136_2021_2846_MOESM1_ESM.docx]

**SUPPLEMENT to Vanier A. et al. Response shift in patient-reported outcomes: definition, theory, and a revised model, Quality of Life Research**

**Supplementary eTable 1. Extant Definitions and Models of Response Shift**

| **Authors** | **Definition** |
| --- | --- |
| Golembiewski (1976) | **Background:**  This definition came up in a paper about experimental designs in management sciences when the outcome of interventions is assessed by measuring within-individual changes over time through self-reports. Golembiewski proposed this conceptual typology of change to expand on the dilemma emphasized by Bereiter in 1963 (Bereiter C (1963) Some persisting dilemmas in the measurement of change. In C.W.Harris (Ed.), *Problems in measuring change*. Madison, WI: University of Wisconsin Press) about the appropriate attribution of observed change in scores to the individual rather than to a change in the structure of the scale of the test.  **Definition:**   - **Alpha change α**: “a variation in the level of some existential state, given a constantly calibrated measuring instrument related to a constant conceptual domain” [p. 134], which occurs during a relatively fixed system or state with discrete and constant intervals. This will later be equated to “true change” in the response shift literature in health sciences. - **Beta change β**: “a variation in the level of some existential state, complicated by the fact that some intervals of the measurement continuum associated with a constant conceptual domain has been recalibrated” [p. 135]; i.e., a recalibration of intervals within a stable dimension of reality. This will later be equated to recalibration in the response shift literature in health sciences. - **Gamma change γ**: “a redefinition or reconceptualization of some domain, a major change in the perspective or frame of reference within which phenomena are perceived and classified, in what is to be taken relevant in some slice of reality” [p. 135]; a change from one state to another. This will later be split into reprioritization and reconceptualization in the response shift literature in health sciences.   **Forms of response shift:**  This conceptualization provides an explicit definition of “true change” as alpha change.  Recalibration is a distinct form of change (referred to as beta change).  Reprioritization and reconceptualization are not viewed as two different forms but rather as the same concept, referred to as gamma change.  In terms of detection and measurement, Golembievski in 1979 posited that gamma change and beta change, respectively, must be ruled out or controlled for before alpha change can be detected as changes in outcome scores lose their meaning if the construct itself and/or the internal standards of measurement have changed. |
| Howard et al. (1979) | **Background:**  Historically, the term “response shift” was introduced by Howard et al. in 1979 in the field of educational training. Their aim was to experimentally assess the efficacy of various training interventions, such as improving leadership and performance appraisal or reducing dogmatism.  The typical approach to assess training interventions involves collecting pretest and posttest data on subjects exposed to the intervention and comparing them with an appropriate control group. In line with usual assumptions on self-report instruments, Howard et al. have posited that for pre-test and post-test scores to be comparable, a common metric has to exist between the two sets of scores. Thus, in the case of self-report, researchers assume subjects have an internalized perception of their level of functioning and this internalized standard would not change from one testing to the next.  Nonetheless, Howard et al. published what they called “a somewhat paradoxical finding”. In a study many subjects reported a higher level of dogmatism after a dogmatism-reducing training despite their own and therapists’ perception that training had been beneficial. They interpreted these results as evidence that the intervention had the ability to improve one’s insight or awareness of his/her own level of dogmatism and that this led to a change of one’s internal standard of measurement over time. Therefore, Howard et al. hypothesized that whenever such a shift occurs, conventional pre-test/post-test self-reports are unable to accurately gauge treatment effect, because the pre-test measurement was inaccurate. They developed a new experimental design, incorporating a retrospective self-assessment of pretest level (also called “then-test”) immediately after post-test assessment.  **Definition:**  The difference between the then-test and the post-test assessment was hypothesized to assess more accurately intervention induced changes regarding the concept of interest. Response shift was operationalized as a significantly larger mean difference between pre-test and then-test self-report ratings in the experimental group than in the control group.  **Forms of response shift:**  Response shift is equated to scale recalibration. Reprioritization and reconceptualization are not addressed. |
| Sprangers & Schwartz (1999) | **Background:**  In 1999, Sprangers & Schwartz translated the notion of response shift into the field of health-related QoL. They believed that response shift would be relevant for the interpretation of some “paradoxical and counter-intuitive” findings like reporting of stable QoL by patients with a life-threatening disease, or discrepancies between clinical measures of health and patients’ own evaluations of their health.  **Definition:**  “A change in the meaning of one's self-evaluation of a target construct as a result of:   1. a change in the respondent's internal standards of measurement (scale recalibration in psychometric terms); 2. a change in the respondent's values (i.e. the importance of component domains constituting the target construct); 3. a redefinition of the target construct (i.e., reconceptualization)” (page 1508)   **Model** (Figure from Sprangers, M. A. G., & Schwartz, C. E. (1999). Integrating response shift into health-related quality of life research: a theoretical model. *Social Science & Medicine*, *48*(11), 1507–1515)**:**  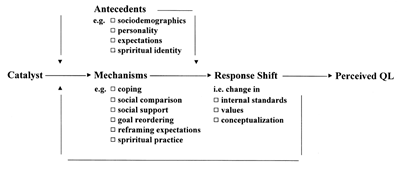  The model was designed to address relationships between five major components, including:   1. catalyst: a salient event leading to a change in the respondent’s health status; 2. antecedents: stable or dispositional characteristics of the individual. They have both direct and indirect effects on potentiating RS. They affect the kind of mechanisms engaged in, the magnitude and type of response shift; 3. mechanisms: behavioral, cognitive and affective processes to accommodate the catalyst, such as the use of coping strategies or upward or downward social comparisons; 4. perceived QoL: a multi-dimensional concept including at least three broad domains (i.e., physical, psychological and social functioning) [3].   The feedback loop included in the model illustrates that the process is thought as iterative and dynamic: perceiving a suboptimal QoL may lead the individual to reinitiate established or new mechanisms.  According to Sprangers & Schwartz, response shift was isolated both from mechanisms and perceived QoL as it conceptualizes aspects that are likely to help understanding observed changes in QoL over time. The isolation of response shift was therefore conceived as a pragmatic approach to explain some aspects of changes in QoL.  **Forms of response shift:**  In this conceptualization, Sprangers & Schwartz do not address how the three forms of response shift are distinct from one another or interconnected. Reprioritization was isolated from reconceptualization to “highlight its importance and emphasize the need to measure it carefully”. |
| Rapkin & Schwartz (2004; 2019) | **Background:**  In 2004, Rapkin & Schwartz extended the theoretical model proposed by Sprangers & Schwartz (1999). They addressed the circularity inherent in that model caused by response shift not being sufficiently differentiated from both mechanisms and outcomes. Therefore, the concept of response shift overlapped with the mechanisms leading to response shift and the outcome affected by response shift.  **Model (**Figure from Rapkin, B. D., & Schwartz, C. E. (2004). Toward a theoretical model of quality-of-life appraisal: Implications of findings from studies of response shift. *Health and Quality of Life Outcomes*, *2*, 14)**:**  To distinguish mechanisms and outcomes from response shit, response shift is considered to be an epiphenomenon occurring when there is a change in appraisal. Rapkin & Schwartz posited that any response to a QoL item can be understood as a function of an appraisal process. They suggested four cognitive processes involved in responding to an item which are:   1. frame of reference, encompassing categories of experiences or events that individuals consider relevant for the completion of a particular item; 2. sampling strategy to extract relevant categories within a frame of reference (e.g. in assessing pain an individual can sample “recent instances of pain” or “times when pain interfered with my activities”); 3. standard of comparison as a reference point to evaluate the specific experiences sampled (e.g. pain experiences may be compared to “worst pain I’ve ever had” or to “what my doctor told me to expect”); 4. combinatory algorithm combining the evaluations into a summary appraisal (e.g., using different subjective weights or relative importance of different experiences to arrive at an answer).   Thus, response shift can be equated neither to mechanisms, nor to the outcome (observed scores). Rather, it is an effect triggered by a catalyst, via mechanisms, mediated through a change in appraisal, leading into changes in observed QoL scores that cannot be explained by standard influences.  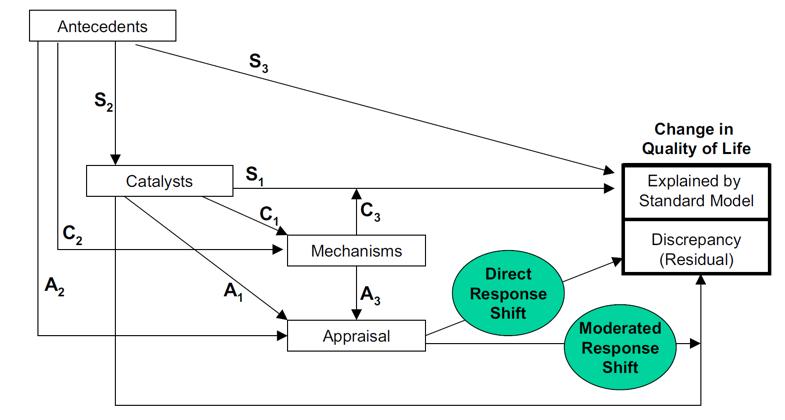  In their 2019 paper, Rapkin & Schwartz posit “that three specific  conditions had to be met for a response shift to have taken place:  • a change in cognitive appraisal of QoL …;  • a discrepancy between the individual’s observed QoL  rating compared to the rating that the individual would  have otherwise made (i.e., unexpected change);  • the ability of change in cognition to explain the unexpected  change in QoL, even after taking into account  any relevant changes in physical health state, treatments,  supports and social determinants.”.  **Forms of response shift:**  In this model, response shift results from a change in the way people appraise the target construct over time.  The three forms of response shift proposed by Sprangers & Schwartz were related to the aforementioned appraisal processes:   1. change(s) in the frame of reference relate to reconceptualization, 2. change(s) in the sampling strategy or the combinatory algorithm relate to reprioritization; 3. change(s) in the standard of comparison relate to recalibration. |
| Oort (2005) | **Background:**  This definition was a first attempt to propose a formal (mathematical) definition of the response shift effect in order to alleviate lack of clarity, especially in the context of detecting response shift using latent variables measurement theories.  **Definition:**  Following the definition of Mellenbergh in 1989 of an unbiased measurement by means of the principle of conditional independence (PCI), response shift is defined as a special case of violation of the PCI.  The measurement of a set of variables A (e.g., physical health, mental health) by a set of variables X (e.g., the scales of a self-report questionnaire) is unbiased with respect to a set of variables V (e.g., disease, treatment, gender, age, ethnicity) if the PCI holds, that is, if  $f_{1}\left( X \vert A=a,V=v \right)=f_{2}\left( X \vert A=a \right)$  for all values a and v of variables A and V, where f_1_ is the conditional distribution function of X given a and v, and f_2_ is the conditional distribution function of X given a If the PCI does not hold, the measurement of A by X is biased by V. X, A and V may be measured on the nominal, ordinal, interval, or ratio level, they may be latent or manifest, and their interrelationships may be linear or nonlinear. Response shift may be considered as a violation of the PCI with variable V being the time of measurement (Definition 1). This definition does cover response shift but it may be too broad. It includes all sources of bias between measurements, among which response tendencies that Sprangers and Schwartz would like to exclude as response shifts. They specifically describe respondents’ response shifts as a consequence of a change in internal standards (recalibration), in values (reprioritization), or in understanding of what is measured (reconceptualization). Therefore, response shift should perhaps be more narrowly defined as a violation of the PCI with recalibration, reprioritization and reconceptualization substituted for V (Definition 2). Even more strict, the response shift label might be reserved for cases in which bias is brought about by a catalyst (e.g., a health state change) substituting V by interactions of recalibration, reprioritization and reconceptualization with the presence of a catalyst (Definition 3).  **Forms of response shift:**  This definition does not make any assumptions about a hierarchical connection between the different forms of response shift. |
| Oort, Visser & Sprangers (2009) | **Background:**  This definition is an extension of the previous one. Its purpose is to provide a broad formal (mathematical) definition of response shift. It introduces two perspectives in order to address an ongoing debate in the response shift literature between a view of response shift as an effect that is mainly measurement bias threatening the internal validity of assessing change over time in PROM scores, and a view seeing response shift as a manifestation of psychological adaptation to illness, and therefore as an effect explaining part of the actual change in a targeted construct.  **Definition:**  The framework of this definition is, as in the previous one, the principle of conditional independence stated by Mellenbergh in 1989. But, an additional perspective (the conceptual perspective) is added.  Measurement perspective:  Response shift can be formally defined as measurement bias (a violation of the PCI if this does not hold: $f_{1}\left( X \vert A=a,V=v \right)=f_{2}\left( X \vert A=a \right)$), with X representing the respondents’ observed scores on repeatedly administered tests, A representing the true attribute values of the respondents at the times of measurement, and V being the time of measurement itself or other variables with effects on X that vary with time of measurement.  In this perspective, response shift is viewed as an effect altering the relationships between A and X. Therefore, here, it is the measurement model between A and X that varies by the presence of V.  Conceptual perspective:  Response shift can be formally defined as explanation bias (a violation of the PCI if this does not hold: $f_{1}\left( X \vert A=a,E=e \right)=f_{2}\left( X \vert A=a \right)$), with A representing (true) attribute values on repeated test administrations, E representing values for acknowledged explanatory variables (causes or predictors) of A, and V being the time of measurement itself or other variables with effects on A that vary with time of measurement.  In this perspective, response shift is viewed as an effect explaining the change in the value of A over time. Change in A value cannot be fully determined by standard explanatory variables E, but also by other variables V (e;g. psychological mechanisms engaged to psychologically adapt to the illness explaining part o the change in the targeted construct, such as coping mechanisms or social comparison).  **Forms of response shift:**  This definition does not make any assumptions about a hierarchical connection between the different forms of response shift. |

**SUPPLEMENT to Vanier A. et al. Response shift in patient-reported outcome measures: definition, theory, and a revised model, Quality of Life Research**

**Supplementary eTable 2. Patients' quotes illustrating the different components of the model**

| **Quotes** | |
| --- | --- |
| **Antecedents** | |
| **Personal factors** | 1. I always faced challenges head on. And so I would face this one the same way. Determination has always been part of who I am, and adversity seemed somehow to make me stronger^1^ 2. ‘My self-confidence at the beginning was absolutely battered to death and since I commenced working again from home last year I feel half alive again. And it has picked me up. I feel that I’ve got a purpose’.^2^ 3. I tried to think about what I could do the rest of my life even though I am on dialysis. Well, I just made up my mind and I’m a determinate person…I’ve always accepted things…I believe what the doctors tell me and well if that’s gonna be what it is, that’s what it is and nothing that you can do about it.^3^ 4. ...it could be a lot worse...some guys in wheelchairs they confine themselves away from people...But I’m not that type...I still knew who I was in the wheelchair...not like I’m living in it, I’m just using it.^4^ |
| **Impairments, activity limitations and participation restrictions** | 1. ‘From lying in bed, being unable to move, someone having to help with bladder and bowel routines, someone needing to turn me every 2 h, to being able to sit in a wheelchair, to eating on my own with an assistive device, I realized I was not going to be a helpless, dependent person. Once I was given options of resuming sexual activity, being able to drive, there was no turning back.’^5^ 2. … That’s the most devastating thing really, the fact that nothing is the same, you know, even a simple thing like walking up the lane, I have to plan it and think about it, work out whether I am capable of doing it.^6^ 3. “First I walked to the golf course, then around it collecting golf balls but when I played nine holes I knew I had made it”^7^ |
| **Catalyst** | |
| **Catalyst** | 1. ‘You say the initial diagnosis was really just a confirmation of what you knew. But, tears came when you lost your hair. Somehow, that makes it real. Knowing it and yet for most women having breast cancer you’re not physically disabled, you’re not physically sick, it’s the treatment that does it. Not the illness. And losing the hair is something that makes it concrete and real and inescapable.’^8^ 2. I think that’s the only, probably the only real loss that I couldn’t do anything about immediately because I had already experienced a loss that made it real to me in February because I took both my breasts off.^8^ 3. When you lose your health and you’re not well…having the whole world is not worth it!^9^ 4. I think because I was very in denial and it was a big shock when [consultant] said ‘I think you need to go on the transplant list [..] all my life I’d always ‘ah I’m alright, I’m fine’ and then it was realising how serious my condition had actually got.^10^ 5. “It makes me feel frightened so to me, it would always be in the back of my mind that I’ve had this diagnosis, it might come back again”^11^ 6. “I never thought I’d look so bad. My eyes became red. My hair fell out. I’d grab pieces of hair. When all your hair goes, you lose your masculinity.”^12^ 7. I never experienced 3 months to go so slowly. Before the disease, I was busy in my job, my studying, but now suddenly my life was stopped. The disease completely changed the direction of my life.^13^ |
|  | |
| **Mechanisms** | |
| **Adaptation - adaptive** | 1. I may just say that you simply have to readjust, and live in a completely different way, you have to constantly keep in mind that you can’t do too much and you always have to adapt yourself in relation to time. You simply have to readjust and rethink in life.^14^ 2. ‘My deficits remain; but I have learned to live relatively peaceful with them, integrating them into my being and managing to enjoy a life which is still rich and active. It is not my previous life. Most people live once; I have had the opportunity to live twice.’^15^ 3. “It took me a while to adjust to the loss of status but now that I have retired I feel much stronger, work is no longer my life, I am happier and no worse off”^7^ 4. One can adapt more than one thinks. The first time you hear about it, you get a terrible shock, but you keep on adapting. Things you consider terrible at first don’t seem so terrible later on. Why does it work this way? Maybe because the mind is compliant and maybe also because you’re just happy to be alive. In the beginning you don’t put up with many complaints. That changes tremendously in time.^16^ 5. The reality is that you’ve got to adjust to it and accept that you are not that person before the diagnosis, really.^17^ |
| **Adaptation - maladaptive** | 1. …my life really is over …it happened overnight and you didn’t have time to think about it …it died overnight …everything ... my job, my voice, my friends, my social life. I don’t go …anywhere! …if I do I go …like my children ’ s birthdays, …I do the cooking; you don’t have to talk to anyone …you are just part of the furniture and …meld into the background.^18^ 2. ‘I have found that in general the public are sort of ignorant about dealing with disabilities, they don’t have the time, they couldn’t be bothered and you sort of get the feeling you are left aside by the roadway. Frustrating stuff…I have changed my attitude to life, completely. I tell you what, what is important for me that counts and the rest of the things they can get stuffed, from my point of view, I couldn’t care less and that’s why she [his wife] says I have become more aggressive.^2^ 3. I said to Dr X, I’ve lost my old nature, I’m not myself… I’m somebody else. No sense of humour, no nothing.^19^ 4. The frustration – and I’ve always done these jobs and now I can’t. I do get irritable sometimes, I’m too ambitious, I’d like to be my old self [our emphasis; annoyed by inactivity–physical limits]. I get despondent when you see other people doing things you used to.^19^ 5. I have been working hard all my life…Now that my children are grown up, I’m sick…I feel deprived of life, empty, and worthless. I always had a key role in the family and supported others…But now I need help and support myself.^9^ 6. ‘I struggle daily to do my job and be the person I used to be. I still, after 2 years, am trying to redefine myself’^15^ |
| **Post-traumatic growth** | 1. While I don’t recommend cancer as a life encounter, my experience is along with the devastation it wreaks there’s a positive side to the experience at least for those of us with supportive family and friends. Being thankful for and appreciative of good family, good friends, and good doctors must be balanced alongside the associated hardships.^20^ 2. Cancer is part of my personal ministry to let others know having cancer can be a blessing regardless of the outcome^20^ 3. ‘It might sound strange but getting an acoustic neuroma was one of the best things that could have happened to me’.^2^ 4. Before I was just living. There was no meaning to life … But now I know my time is limited. I need to do things. I need to have an impact on some things. […] before I wasn't doing what I wanted, I was living for others. Right now I am living for me, me, me, me. Myself … I found who I am. I found out who I am^21^ 5. [Surviving cancer] confirmed to me that we are watched over, because there were many miracles that happened while I was sick, and I should have died at least three times. I don't know. It was a good experience for that overall. I mean it was awful, but it was also good.^22^ 6. ...you start looking through a different window. It’s not what it was...and we’re going to be in misery for a long time...Took me two years. I beat my head off shit for two years. Didn’t happen overnight^4^ |
| **Transformative learning** | 1. …transformative is a very good word …almost a sea change in ... in the sense that it teaches you about yourself. ... who you really are when everything else is stripped away and that is really a valuable experience …and the additional richness that it’s put into my life …I’ve become much more mellow with life.^18^ 2. ‘It’s helpful because I know that okay these people, you know are different to me, but they’re also leading a life and they’ve had the same sort of problems as I have and they’ve got over it, yeah it helps’^23^ 3. I remember the nurse saying to me ‘You’ll never walk again. Don’t try to move your legs. You’re wasting your time’. She was right because I never moved them then and haven’t moved them since. That brought it home to me. You’ve got to get on with your life as it is and from then on I did. I know your relatives say ‘You’ll be all right. We’ll sort it out’, but it doesn’t happen, but that is bad for the patient.^24^ 4. To me, this group was a reinforcement because I had more strength, since you can see you are living the same things others are too. You do not feel lonely, and you see that difficulty can be overcome.^25^ 5. Am I more resigned to the fact that I’m still here and I don’t care. You know, I am in a wheelchair and that is it. That’s the most important thing to me is that I’m alive, and to walk again does not bother me. I’ve got my wife, I’ve got my kids, I’ve got my family around me, I’ve got everything that I need. I’ve got a good life, quality of life. It’s changed in 12 months, don’t get me wrong, it has, a lot, and I’m still learning, I’m still coming to terms with it. Still coping with things and new things […] but […] I am quite happy the way my life is, because my life’s more important.^24^ 6. “You move from a sense of helplessness to a condition in which you feel you can control your illness.”^25^ 7. I am the same person but now I have different ways to see everything. ^26^ |
| **Non-uniform response shift** | 1. I can explain it like this. In the beginning you feel totally relieved that the cancer has disappeared and actually you take everything that comes along as part of the deal. But in the end you start feeling the annoyance of urinary leakage and diminished erection.^16^ |
| **Theories of response shift** | |
| **Theory of how response shift occurs** | |
| **Recalibration/Appraisal** | 1. “My health is unstable. When I come home from chemotherapy I feel like a “5’, I feel nauseated right after the treatment. But other than that, I do not feel sick. So I cannot say my health is a ‘5’. On the other hand, it isn’t a ‘7’ either, because I only feel like a 7 in the last week before the next chemo. So I will opt for a ‘6’, right in between.^27^ 2. “I think that I’ve said a little last time, it felt a little, but I shift my limit”^28^ 3. “I told you that I was really tired then, but compared to how I’m feeling now, it was then just a piece of cake”^28^ 4. I didn’t consider the consequences of prostate cancer treatment much when I answered questions about my health, because… after all those years I’ve got more or less used to them. […] That’s just a matter of acceptance.^16^ 5. “I already told you that I would change my standards”^28^ |
| **Reconceptualization/Appraisal** | 1. “So that’s what to me the new norm, or normality is, it’s a new norm about living with your cancer but the history that you’ve just had”^11^ 2. I was in hospital with people that were really bad, that couldn’t walk properly, couldn’t dress, couldn’t feed themselves, couldn’t go to the toilet, had to wear liners all the time. Em ... I could have been so much worse. And each day when I see a slight improvement, and I do every day, I’m so thankful.^29^ 3. ‘‘We could be a lot worse off. But then sometimes we don’t think that way unless when something goes wrong and then you go all this and that’’^30^ 4. She told me, […] ‘I'm positive as well’ but she was looking good. I said ‘Oh my god’ Then I knew my life because, if a person like her, looking good as she is, then I should do something and stop moaning about myself, so I stopped.^21^ 5. “When I saw the other people on the course who were far worse than me and they were dealing with things, I realized I wasn’t so badly off.”^31^ 6. ‘‘It is hard but some days I get a bit teary and that but then when I look around [I think] I am lucky.’’^32^ |
| **Reprioritization/Appraisal** | 1. Yes. I’m lucky because I know when I need to go to the toilet so I don’t have to wear a bag or anything like. It’s worse than being in a wheelchair, not having control of your functions. I would quite happily be in a wheelchair because I can still do what I want to do, drive the car and everything, but not have control of your bodily functions is the worst part of the whole thing.^21^ 2. And I think that things in life that were important to you or you thought were important, aren’t as important. I want to stay alive, I want to see my kid get married and have grandchildren. There is a whole bunch of things that you think about that you want to happen. Whatever I’ve got to live with, I’ll live with, and that’s all.”^33^ 3. I lost my hair during the second course of treatments. But the consultant did say, ‘You are going to lose your hair’. And I says, ‘Oh okay’. But it’s growing back a bit now. But I will lose it again, but that’s okay. I’m not bothered this time. I can live without hair, it doesn’t bother me, you know.^17^ 4. I was a trained dancer before my injury and my heart wanted to follow that profession. Through the process of hoping for a better future, I moved towards acceptance of the mobility limitations due to my injury. I re-evaluated my priorities. I started setting targets and goals. When those targets kept getting met, I reaffirmed the faith in my abilities^5^ |
| **Theory of why response shift occurs** | |
| **Cognitive Homeostasis / Set point theory** | 1. rather than think I wish I could be doing this, I wish I could be doing that., I don’t, I just say right, I’m not going to be able to do this, I’ll shrink it…so you cope that way really.^23^ 2. “I want to stay alive, I want to see my kid get married and have grandchildren. There is a whole bunch of things that you think about that you want to happen. Whatever I’ve got to live with, I’ll live with, and that’s all.” 3. ‘My deficits remain; but I have learned to live relatively peaceful with them, integrating them into my being and managing to enjoy a life which is still rich and active. It is not my previous life. Most people live once; I have had the opportunity to live twice.’^15^ |

**Notes**

References belonging to Supplementary eTable 2 follow below.

Methods: A systematic search was conducted on three databases: MEDLINE, PsycINFO and EMBASE. Response shift and related keywords (e.g., reprioritization, adjustment, recovery) were used. For studies to be included, the content had to involve the change in the perception of the experience of living with a health condition. Study selection was conducted by NO and NM. Quotes from included articles were then mapped onto the different processes of the model.

**References belonging to Supplementary eTable2 Patients' quotes illustrating the different components of the model**

1. Curtis R, Groarke A, McSharry J, Kerin M. Experience of breast cancer: Burden, benefit, or both? *Cancer Nursing*. 2014;37:E21-30

2. Brooker J, Burney S, Fletcher J, Dally M. A qualitative exploration of quality of life among individuals diagnosed with an acoustic neuroma. *British Journal of Health Psychology*. 2009;14:563-578

3. Elliott BA, Gessert CE, Larson PM, Russ TE. Shifting responses in quality of life: People living with dialysis. *Quality of Life Research*. 2014;23:1497-1504

4. Rohn EJ, Tate DG, Forchheimer M, DiPonio L. Contextualizing the lived experience of quality of life for persons with spinal cord injury: A mixed-methods application of the response shift model. *Journal of Spinal Cord Medicine.* 2018

5. Parashar D. The trajectory of hope: Pathways to find meaning and reconstructing the self after a spinal cord injury. *Spinal Cord*. 2015;53:565-568

6. Alaszenwski A, Alaszewski H, Potter J. The bereavement model, stroke and rehabilitation: A critical analysis of the use of a psychological model in professional practice. *Disability and Rehabilitation*. 2004;26:1067-1078

7. Appleton L, Goodlad S, Irvine F, Poole H, Wall C. Patients' experiences of living beyond colorectal cancer: A qualitative study. *European Journal of Oncology Nursing*. 2013;17:610-617

8. Ferrell BR, Grant MM, Funk B, Otis-Green S, Garcia N. Quality of life in breast cancer survivors as identified by focus groups. *Psycho-Oncology*. 1997;6:13-23

9. Abdi F, Daryani NE, Khorvash F, Yousefi Z. Experiences of individuals with liver cirrhosis: A qualitative study. *Gastroenterology nursing : the official journal of the Society of Gastroenterology Nurses and Associates*. 2015;38:252-257

10. Anderson SM, Wray J, Ralph A, Spencer H, Lunnon-Wood T, Gannon K. Experiences of adolescent lung transplant recipients: A qualitative study. *Pediatric Transplantation*. 2017;21 (3) (no pagination)

11. Appleton L, Flynn M. Searching for the new normal: Exploring the role of language and metaphors in becoming a cancer survivor. *European journal of oncology nursing : the official journal of European Oncology Nursing Society*. 2014;18:378-384

12. Baker F, Zabora J, Polland A, Wingard J. Reintegration after bone marrow transplantation. *Cancer Practice*. 1999;7:190-197

13. Fan SY, Eiser C. Illness experience in patients with hepatocellular carcinoma: An interpretative phenomenological analysis study. []. *European Journal of Gastroenterology & Hepatology.* 2011;01

14. Ostman M, Jakobsson Ung E, Falk K. Continuity means "preserving a consistent whole"--a grounded theory study. *International journal of qualitative studies on health and well-being*. 2015;10:29872

15. Levack WM, Kayes NM, Fadyl JK. Experience of recovery and outcome following traumatic brain injury: A metasynthesis of qualitative research. *Disability & Rehabilitation*. 2010;32:986-999

16. Korfage IJ, Hak T, de Koning HJ, Essink-Bot M-L. Patients' perceptions of the side-effects of prostate cancer treatment--a qualitative interview study. *Social Science & Medicine*. 2006;63:911-919

17. Vas S, Povey R, Clark-Carter D. 'I would describe myself as a deformed troll': Using interpretative phenomenological analysis to explore body image struggles among palliative care patients. *Palliative Medicine*. 2019;33:232-240

18. Bickford J, Coveney J, Baker J, Hersh D. Living with the altered self: A qualitative study of life after total laryngectomy. *International Journal of Speech-Language Pathology*. 2013;15:324-333

19. Dowswell G, Lawler J, Dowswell T, Young J, Forster A, Hearn J. Investigating recovery from stroke: A qualitative study. *Journal of clinical nursing*. 2000;9:507-515

20. Leal I, Milbury K, Engebretson J, Matin S, Jonasch E, Tannir N, et al. Interconnection: A qualitative analysis of adjusting to living with renal cell carcinoma. *Palliative & supportive care*. 2018;16:146-154

21. Dibb B, Kamalesh T. Exploring positive adjustment in hiv positive african women living in the uk. *AIDS Care - Psychological and Socio-Medical Aspects of AIDS/HIV*. 2012;24:143-148

22. Zamora ER, Yi J, Akter J, Kim J, Warner EL, Kirchhoff AC. 'Having cancer was awful but also something good came out': Post-traumatic growth among adult survivors of pediatric and adolescent cancer. *European journal of oncology nursing : the official journal of European Oncology Nursing Society*. 2017;28:21-27

23. Beeken RJ, Eiser C, Dalley C. Health-related quality of life in haematopoietic stem cell transplant survivors: A qualitative study on the role of psychosocial variables and response shifts. *Quality of Life Research*. 2011;20:153-160

24. Dibb B, Ellis-Hill C, Donovan-Hall M, Burridge J, Rushton D. Exploring positive adjustment in people with spinal cord injury. *J Health Psychol*. 2014;19:1043-1054

25. Borghi M, Bonino S, Graziano F, Calandri E. Exploring change in a group-based psychological intervention for multiple sclerosis patients. *Disability and rehabilitation*. 2018;40:1671-1678

26. von Vogelsang A-C, Wengstrom Y, Svensson M, Forsberg C. Transitional experiences in patients following intracranial aneurysm rupture. *Journal of Clinical Nursing*. 2014;23:1263-1273

27. Taminiau-Bloem EF, van Zuuren FJ, Koeneman MA, Rapkin BD, Visser MR, Koning CC, et al. A 'short walk' is longer before radiotherapy than afterwards: A qualitative study questioning the baseline and follow-up design. *Health & Quality of Life Outcomes*. 2010;8:69

28. Westerman MJ, The A-M, Sprangers MA, Groen HJ, van der Wal G, Hak T. Small-cell lung cancer patients are just 'a little bit' tired: Response shift and self-presentation in the measurement of fatigue. *Quality of Life Research*. 2007;16:853-861

29. Pringle J, Drummond JS, McLafferty E. Revisioning, reconnecting and revisiting: The psychosocial transition of returning home from hospital following a stroke. *Disability and rehabilitation*. 2013;35:1991-1999

30. Byrne-Davis LMT, Bennett PD, Wilcock GK. How are quality of life ratings made? Toward a model of quality of life in people with dementia. *Quality of Life Research*. 2006;15:855-865

31. Osborne RH, Hawkins M, Sprangers MAG. Change of perspective: A measurable and desired outcome of chronic disease self-management intervention programs that violates the premise of preintervention/postintervention assessment. *Arthritis Care and Research*. 2006;55:458-465

32. Bartley E, White J, Janssen H, Spratt NJ, Pollack M. Exploring the experience of stroke rehabilitation following exposure to an enriched environment. *International Journal of Stroke*. 2012;1):44

33. Neuman HB, Park J, Fuzesi S, Temple LK. Rectal cancer patients' quality of life with a temporary stoma: Shifting perspectives. *Diseases of the Colon and Rectum*. 2012;55:1117-1124

**SUPPLEMENT to Vanier A. et al. Response shift in patient-reported outcome measures: definition, theory, and a revised model, Quality of Life Research**

**Supplementary eText 1. Some examples on how to empirically test (parts of) the revised model of response shift.**

As described in the paper, we have not specified how the depicted entities are operationalized nor how these are mathematically linked. Based on a literature review and expert consensus [1],we provide a conceptual model that guides testing of response shift phenomena, describing the conditions that need to be met and alternative explanations that need to be excluded when investigating response shifts. These are deliberately formulated in a general form to allow researchers to use the appropriate methods in their chosen methodological framework.

As studies testing the entire Sprangers and Schwartz model [2] on empirical data were already rare (one example is Visser et al. [3]), we anticipate that support for (or refutation of) this revised model can only be found across multiple studies, employing a variety of methods. To define a full research agenda is beyond the scope and intention of our work. Nevertheless, in the following we highlight approaches that we feel are particularly important.

1. While our work extends previous definitions of response shift, one of the key assumptions remains that response shift occurs as a consequence of “a change in the meaning of one’s self evaluation of a target construct”. Response shift arises from particular adaptation processes within individuals and this is one of the key distinctions from processes that offer alternative explanations for the phenomenon (see main manuscript, Table 1). The revised model is based on extensive evidence including qualitative methods and these will remain one of the avenues to further understanding and theory building regarding response shift (e.g., semi-structured interviews and conceptual analysis of the verbatim responses; e.g., supplementary eTable2). The revised model can be used to develop longitudinal qualitative studies to investigate such adaptation processes, thereby focusing on particular paths, selected antecedents, catalysts or mechanisms.
2. The importance of individualized patient reported outcome measures (PROMs) for understanding adaptation and recovery processes has been stressed repeatedly in response shift research (e.g. patient generated index) [4]. A range of these indices have been used to gather both ideographic quantitative information about the investigated outcome domain(s) as well as qualitative information about the use and meaning of the PROM. These measures will continue to contribute essential information as they can be applied within the frameworks of a range of theories of *why response shift occurs* and support investigations of a wide range of the depicted pathways in Figure 1.
3. Similarly, the investigation of the role of appraisal in the processes leading to response shifts is an important existing branch of response shift research [5]. The use of interviews and measures capturing appraisal processes (e.g. such as the Brief Appraisal Profile [6]) will therefore remain another key area of evidence generation. As argued in the main manuscript, appraisal theory describes a constellation of pathways of the model of *how response shifts occur* (Figure 1). Therefore, investigations using this framework will remain important empirical tests of the revised model of response shift, especially when researching more than one of these mechanisms at once. Methods are available that can be applied to investigate whether changes in appraisal explain the mismatch between change in the target construct and observed change. These methods will continue to support appraisal-focused research and may serve as examples for developments of approaches to capture processes from other theories of how response shift can occur.
4. Structural Equation Models are a tool that seems a particularly well-suited quantitative approach to test predictions derived from the revised model [7]. A focal area for such studies is investigating the path explaining the occurrence of a response shift as an effect mediated by psychological mechanisms (Figure 1, M2 path). Based on the revised definition, it could be tested if a longitudinal measurement model where mechanism(s) explain both the value of factor scores and the value of manifest variables fits better to a model where mechanisms only explain factor scores (for example King-Kallimanis et al. [8]).
5. Finite mixture models describe a group of statistical approaches that allow to identify subgroups of people (latent classes) where standard statistical approaches assume that the same parameters describe the whole analyzed sample or that the parameters vary by a continuous function or assume shared error terms (e.g., mixed models). An area where this approach is increasingly used is to identify which respondents may have experienced the same response shift pattern. These models have been used to investigate the measurement model paths of Figure 1 in more detail (TC1 1, TC2 1, TC1 4, Me1 1) and the resulting response classes can be used to explore their relationships to antecedents, catalysts and mechanisms [9, 10]. Since the development of structural equation mixture models is currently a growth area in applied statistics, future research may also be able to investigate larger parts of the revised model from a latent mixture perspective.
6. A so far under-utilized approach in response shift research is the use of experimental studies. The revised model presents causal connections between the elements of the response process, across time and how they lead to response shift (vs. alternative explanations of response behavior, see Table 1). Combining the model with theories of why response shift can occur (Figure 1) can support the design of experimental studies that test pathways explicitly. Interventions aimed at understanding disease and recovery processes, may introduce particular coping mechanisms or may have the potential to induce transformative learning more broadly [4], target interactions of antecedents, catalysts and mechanisms (e.g., Figure 1: paths A2, A4, C1, M1, M2). If formulated in terms of the revised model, these can result in strong empirical tests, by randomizing to such interventions as well as potentially generating new evidence about the effectiveness and mechanisms of such interventions. Extending this argument, adopting a causal modelling perspective on response shift and more frequently leveraging prospective research designs with specific predictions regarding the occurrence of response shifts (e.g., through planned analyses in public protocols, analyses plans or registered reports), could further contribute to the existing evidence base and our understanding of response shifts.

**REFERENCES**

1. Sprangers, M. A. G., Sajobi, T. T., Vanier, A., Mayo, N. E., Sawatzky, R., Lix, L., … and the Response Shift - in Sync Working Group. (2021). Response shift in results of patient-reported outcome measures: A commentary to the Response Shift - in Sync Working Group Initiative. *Quality of Life Research*, *Online ahead of print*.

2. Sprangers, M. A. G., & Schwartz, C. E. (1999). Integrating response shift into health-related quality of life research: a theoretical model. *Social science & medicine*, *48*(11), 1507–1515.

3. Visser, M. R. M., Oort, F. J., Lanschot, J. J. B., Velden, J., Kloek, J. J., Gouma, D. J., … Sprangers, M. A. G. (2012). The role of recalibration response shift in explaining bodily pain in cancer patients undergoing invasive surgery: an empirical investigation of the Sprangers and Schwartz model. *Psycho-Oncology*, n/a-n/a. https://doi.org/10.1002/pon.2114

4. Mayo, N. E. (2019). Appraisal as a unifying theory of response shift: continuing the conversation. *Quality of Life Research* *28*(10), 2635–2636. https://doi.org/10.1007/s11136-019-02270-1

5. Rapkin, B. D., & Schwartz, C. E. (2019). Advancing quality-of-life research by deepening our understanding of response shift: a unifying theory of appraisal. *Quality of Life Research* *28*(10), 2623–2630. https://doi.org/10.1007/s11136-019-02248-z

6. Rapkin, B. D., Garcia, I., Michael, W., Zhang, J., & Schwartz, C. E. (2017). Development of a 5practical outcome measure to account for individual differences in quality-of-life appraisal: the Brief Appraisal Inventory. *Quality of Life Research*. https://doi.org/10.1007/s11136-017-1722-2

7. Oort, F. J. (2005). Using structural equation modeling to detect response shifts and true change. *Quality of Life Research*, *14*(3), 587–598.

8. King-Kallimanis, B. L., Oort, F. J., Visser, M. R. M., & Sprangers, M. A. G. (2009). Structural equation modeling of health-related quality-of-life data illustrates the measurement and conceptual perspectives on response shift. *Journal of Clinical Epidemiology*, *62*(11), 1157–1164. https://doi.org/10.1016/j.jclinepi.2009.04.004

9. Mayo, N. E., Scott, S. C., Dendukuri, N., Ahmed, S., & Wood-Dauphinee, S. (2008). Identifying response shift statistically at the individual level. *Quality of Life Research* *17*(4), 627–639. https://doi.org/10.1007/s11136-008-9329-2

10. Salmon, M., Blanchin, M., Rotonda, C., Guillemin, F., & Sébille, V. (2017). Identifying patterns of adaptation in breast cancer patients with cancer-related fatigue using response shift analyses at subgroup level. *Cancer Medicine*, *6*(11), 2562–2575. https://doi.org/10.1002/cam4.1219

**Appendix Members of the Response Shift –in Sync Working Group**

- Amelie Anota, Department of Human and Social Sciences INSERM UMR 1098, Cancer Care Center Léon Bérard, Lyon, France;
- Oluwagbohunmi Awosoga, Faculty of Health Sciences, University of Lethbridge, Lethbridge, Canada;
- Olawale F. Ayilara, Department of Community Health Sciences, University of Manitoba, Winnipeg, Canada;
- Ruth Barclay, Department of Physical Therapy, University of Manitoba, Winnipeg, Canada;
- Jan R. Böhnke, University of Dundee, School of Health Sciences, Dundee, United Kingdom;
- Anita Brobbey, Department of Community Health Sciences, University of Calgary, Calgary, Canada;
- Cynthia Chauhan, Patient Representative, Wichita, KS, USA;
- Lori Frank, Behavioral & Policy Sciences, RAND Corporation, Arlington, VA, USA;
- Bernice G. Gulek, University of Washington, Harborview Medical Center, Seattle, WA, USA and Washington State University, College of Nursing of Nursing, Spokane, WA, USA;
- Wilbert van den Hout, Medical Decision Making, Department of Biomedical Data Sciences, Leiden University Medical Center, Leiden, The Netherlands;
- A. Cecile J. W. Janssens, Department of Epidemiology, Rollins School of Public Health, Emory University, Atlanta, GA, USA;
- Lene Kongsgaard Nielsen, Department of Haematology, Quality of Life Research Center, Odense University Hospital, Odense, Denmark and Department of Internal Medicine and Cardiology, Regional Hospital Viborg, Viborg, Denmark;
- Jae-Yung Kwon, School of Nursing, University of British Columbia, Vancouver, Canada;
- Oluwaseyi Lawal, Department of Community Health Sciences, University of Calgary, Calgary, Canada;
- Lisa M. Lix, Department of Community Health Sciences, University of Manitoba, Winnipeg, Canada;
- Nancy Mayo, Department of Medicine, Division of Clinical Epidemiology, Center for Outcomes Research and Evaluation (CORE), McGill University, Montreal, Canada;
- Leah McClimans, Department of Philosophy, University of South Carolina, Columbia, SC, USA;
- Sandra Nolte, ICON GmbH, Munich, Germany and Charité – Universitätsmedizin Berlin, corporate member of Freie Universität Berlin, Humboldt-Universität zu Berlin, and Berlin Institute of Health, Medical Department, Division of Psychosomatic Medicine, Berlin, Germany;
- Frans J. Oort, Research Institute of Child Development and Education, University of Amsterdam, Amsterdam, The Netherlands;
- Nikki Ow, School of Physical and Occupational Therapy, Center for Outcomes Research and Evaluation (CORE), McGill University Montreal, Canada;
- Tolulope T. Sajobi, Department of Community Health Sciences and O'Brien Institute for Public Health, University of Calgary, Calgary, Alberta, Canada;
- Richard Sawatzky, School of Nursing, Trinity Western University, Langley, Canada;
- Véronique Sébille, UMR INSERM 1246, SPHERE "methodS in patient-centered outcomes and HEalth ResEarch", University of Nantes, University of Tours, Nantes, France;
- Mirjam A. G. Sprangers, Department of Medical Psychology, Amsterdam University Medical Centers, Research Institute Amsterdam Public Health, Amsterdam, The Netherlands;
- Antoine Vanier, UMR INSERM 1246, SPHERE "methodS in patient-centered outcomes and HEalth ResEarch", University of Nantes, University of Tours, Nantes, France;
- Mathilde G. E. Verdam, Department of Methodology and Statistics, Institute of Psychology, Leiden University, Leiden, The Netherlands.
